# Primaquine Diphosphate, a Known Antimalarial Drug, Blocks Vascular Leakage Acting Through Junction Stabilization

**DOI:** 10.3389/fphar.2021.695009

**Published:** 2021-06-04

**Authors:** Minyoung Noh, Haiying Zhang, Hyejeong Kim, Songyi Park, Young-Myeong Kim, Young-Guen Kwon

**Affiliations:** ^1^ Department of Biochemistry, College of Life Science and Biotechnology, Yonsei University, Seoul, South Korea; ^2^ R&D Department, Curacle Co. Ltd., Seongnam-si, South Korea; ^3^ Vascular System Research Center and Department of Molecular and Cellular Biochemistry, School of Medicine, Kangwon National University, Chuncheon, South Korea

**Keywords:** primaquine diphosphate, endothelial barrier, permeability, USP1, vascular leakage blokcer

## Abstract

Endothelial barrier integrity is important for vascular homeostasis, and hyperpermeability participates in the progression of many pathological states, such as diabetic retinopathy, ischemic stroke, chronic bowel disease, and inflammatory disease. Here, using drug repositioning, we discovered that primaquine diphosphate (PD), previously known as an antimalarial drug, was a potential blocker of vascular leakage. PD inhibited the linear pattern of vascular endothelial growth factors (VEGF)-induced disruption at the cell boundaries, blocked the formation of VEGF-induced actin stress fibers, and stabilized the cortactin actin rings in endothelial cells. PD significantly reduced leakage in the Miles assay and mouse model of streptozotocin (STZ)-induced diabetic retinopathy. Targeted prediction programs and deubiquitinating enzyme activity assays identified a potential mechanism of action for PD and demonstrated that this operates via ubiquitin specific protease 1 (USP1). USP1 inhibition demonstrated a conserved barrier function by inhibiting VEGF-induced leakage in endothelial permeability assays. Taken together, these findings suggest that PD could be used as a novel drug for vascular leakage by maintaining endothelial integrity.

## Introduction

The endothelial barrier maintains vascular and tissue homeostasis (Komarova and Malik, 2010; Park-Windhol and D’Amore, 2016). Vascular permeability is determined by intercellular junctions that create barriers to control the extravasation of plasma (Claesson-Welsh et al., 2020). Endothelial cells are connected by two types of intercellular junctions: adherens junctions (AJs) and tight junctions (TJs) (Bazzoni and Dejana, 2004). Both junctional complexes form pericellular zipper-like structures along the endothelial cell boundaries through the adhesion of distinct adhesive proteins. AJs initiate cell-cell contact and mediate the maturation and maintenance of contact. AJs are composed of vascular endothelial (VE)-cadherin and are related α-, β-, and p120-catenin adhesion complexes (Leach et al., 1993; Lampugnani et al., 1995; Tamura et al., 1998). Several studies have shown that VE-cadherin adhesion is a major adhesion event during vascular development. The degradation of AJs, impaired by the integrity of the VE-cadherin adhesion complex, is the leading cause of tissue edema associated with a wide range of pathological conditions. TJs regulate the pathways around the cells for the movement of ions and solutes between cells. TJs consists of the transmembrane proteins occludin, claudin, and cytoplasmic scaffolding proteins ZO-1, -2, and -3 (Hartsock and Nelson, 2008; Komarova et al., 2017). ZO-1 regulates the cross-interaction between TJs and AJs through intracellular tension and assembly of the VE-cadherin mechanosensory complex. In particular, the pathophysiology of the retina and retinal pigment epithelium membrane is the site of cellular communication and adhesion. AJs and TJs form a composite that is involved in forming a physical barrier, maintaining cell polarity, and preventing intramembrane diffusion between the basal side and apical membrane domains (Shin et al., 2006; Rizzolo, 2007). These junctions dissolve in response to several stimuli, including vascular endothelial growth factor (VEGF) and inflammatory cytokines, such as histamine and bradykinin, allowing the outflow of macromolecules (Claesson-Welsh, 2015). In diseases characterized by excessive vascular permeability (also called vascular leakage), the regulation of junction dynamics is lost, and the junction remains open (Nagy et al., 2008; Claesson-Welsh, 2015).

Reduced barrier function (and increased vascular permeability) is associated with organ dysfunction and can participate in the progression of many pathological conditions, such as diabetic retinopathy (DR), chronic inflammatory disease, and lung injury. Restoration of endothelial barrier integrity under these conditions can significantly delay disease progression (Dejana et al., 2009; Kumar et al., 2009; Rodrigues and Granger, 2015). DR is one of the most common microvascular complications of diabetes mellitus and is the leading cause of blindness in the working-age group (Cheung et al., 2010). The earliest sign of DR is a weakening of the blood-retinal barrier, which leads to a leak in the vessels, followed by retinal edema (Frank, 2004; Shin et al., 2014). VEGF is known to play an important role in blood-retinal barrier breakdown by altering junction integrity and cytoskeletal tissue of endothelial cells, which increases permeability during the pathogenesis of DR (Murata et al., 1995; Weis and Cheresh, 2005; Caprnda et al., 2017). Therapies targeting this early and reversible stage of blood-retinal barrier breakdown remain to be developed. Therefore, to prevent these diseases, treatments that can block vascular leakage are needed.

In this study, we aimed to find a United States Food and Drug Administration (FDA)-approved drug that could be repurposed as a vascular leakage blocker. Primaquine diphosphate (PD) was found to block vascular leakage in endothelial cells through a previously established screening in our laboratory (Maharjan et al., 2011). Primaquine, an 8-aminoquinoline, has been approved by the FDA for the treatment of malaria since 1952 (Hill et al., 2006). However, to date, the anti-permeability properties of PD remain unexplored. Here, we evaluated the therapeutic agent for PD as a vascular leakage blocker and investigated ubiquitin-specific protease 1 (USP1), which is a potential target of PD.

## Methods

### Drug and Inhibitors

FDA-approved drugs (1,018) and PD were purchased from Selleckchem (Houston, TX, United States). PD is a yellow powder that is soluble in water and has a molecular weight of 455.34. SJB2-043 and ML-323, USP1 inhibitors, were purchased from Selleckchem (Houston, TX, United States).

### Cell Culture

Human umbilical vein endothelial cells (HUVECs) were purchased from Lonza (Basel, Switzerland). Cells were grown in 2% gelatin-coated dishes and maintained in medium 199 (Invitrogen, CA, United States) containing 20% fetal bovine serum (HyClone, Tianjin), 1% penicillin/streptomycin, 3 ng/ml basic fibroblast growth factor (R&D system, Minneapolis), and 5 U/mL heparin (Sigma-Aldrich, MO, United States) at 37°C in humidified 5% (v/v) CO_2_ atmosphere. Human retinal endothelial cells (HRECs) were purchased from Cell Systems Inc. (Kirkland, WA, United States). Cells were grown in 2% gelatin-coated dishes and maintained in EC basal medium (EBM-2, CC-3156) containing EGM-2-kit (CC-4176) (Lonza Walkersville, Inc., MA, United States) and 20% fetal bovine serum at 37°C in humidified 5% (v/v) CO_2_. Cell passages between 3 and 6 were used for experiments.

### 3-(4,5-Dimethylthiazol-2-yl)-2,5-Diphenyltetrazolium Bromide Assay

HUVECs were seeded at a density of 3.0 × 10^4^ and 1.0 × 10^4^ cells/well in gelatin-coated 24-well plates and gelatin-coated 96-well plates, respectively, and incubated overnight. Cells were washed and switched to serum-free media and treated with various concentrations of PD. After 48 h, cells were washed, and 0% M-199 containing MTT (0.1 mg/ml) was added, followed by incubation at 37°C for 3 h. The residual MTT was carefully removed, and crystals were dissolved by incubation with dimethyl sulfoxide:ethanol (1:1). Absorbance was measured at 560 nm using spectrophotometry.

### *In Vitro* Vascular Permeability Assay

HUVECs were seeded at a density of 6.0 × 10^4^ cells/well on the luminal side of filters (0.4 μm pore size; Corning) coated with 1% gelatin in 12-well plates. Cells were grown in EC basal medium (EBM-2, CC-3156) containing EGM-2-kit (CC-4176) (Lonza Walkersville, Inc., MA, United States) and 10% fetal bovine serum at 37°C in humidified 5% (v/v) CO_2_. Cells were cultured for 2 days until confluent, and starved in serum-free medium for 2 h and treated with PD (5 µM) for 30 min before induction with VEGF (30 ng/ml; Komabiotech) for 30 min. Transendothelial electrical resistance (TEER) was measured using a chop-stick electrode (World Precision Instruments STX2) with Millicell ERS-2 volt/Ω m (Millipore, MA, United States). The TEER of the cell-free gelatin-coated filters was subtracted from the measured TEER and are given as Ω × cm^2^. Paracellular vascular permeability was also confirmed using fluorescein isothiocyanate (FITC)- dextran fluorescein. FITC-dextran (30 mg/ml; Sigma) was added to the upper compartment. The absorbance of the lower chamber solution was measured at 492 nm (excitation) and 520 nm (emission) using a FLUOstar Omega microplate reader.

### Immunofluorescence Staining of Human Umbilical Vein Endothelial Cells

HUVECs were fixed in 4% paraformaldehyde for 20 min at room temperature and permeabilized in 0.1% Triton X-100 in PBS for 15 min at 4°C. The cells were incubated for 2 h at room temperature with antibodies such as anti-VE-cadherin (1:400, Santa Cruz Biotechnology). The cells were incubated with secondary antibodies conjugated with Alexa Fluor 594 for 1 h at room temperature.

Actin filaments were monitored with rhodamine phalloidin (1:250, Molecular Probes) for 30 min. Cells were mounted using Dako mounting reagent and observed using a fluorescence microscope (Zeiss; ×200) and confocal microscopy (LSM 700 META; Carl Zeiss).

### Western Blot Analysis

HUVECs were washed with cold 1× phosphate-buffered saline and lyzed with 200 μL of RIPA buffer (100 mM Tris-Cl, 5 mM EDTA, 50 mM NaCl, 50 mM β-glycero-phosphate, 50 mM NaF, 0.1 mM Na_3_VO_4_, 0.5% NP-40, 1% Triton X-100, and 0.5% sodium deoxycholate) at 4°C. Lysates were centrifuged at 14,000 rpm for 15 min. Protein samples were separated by electrophoresis on a sodium dodecyl sulphate-polyacrylamide gel and transferred to nitrocellulose membranes. Immunoblotting was performed using antibodies against USP1 (Cell Signaling Technology, Inc., MA, United States) and β-actin (Thermo Fisher Scientific, MA, United States).

### Reverse Transcription Polymerase Chain Reaction

RNA was isolated using Trizol (iNtRON), and RT-PCR was performed using 2× Maxima SYBR Green/ROX qPCR Master Mix (Thermo Scientific, K0221). All results were normalized to GAPDH expression levels.

### Transfection With Small Interfering Ribonucleic Acid

Knockdown of USP1 expression was targeted by using USP1-specific ON-Target SMARTpool siRNA. Parallel transfection with a pool of ON-TARGET plus non-targeting siRNAs served as a negative control. The pools of siRNA sequences were obtained from Dharmacon (Waltham, MA, United States). Lipofectamine *in vitro* transfection reagent (Invitrogen, CA, United States) was used to deliver siRNA into the cell. Transfection was performed according to the manufacturer’s instructions in cell monolayers at 70–80% confluency. Cells were harvested 48 h post-transfection, followed by analysis using both the permeability assay and immunofluorescence.

### Ub-AMC Assay

All deubiquitinating enzyme (DUB) reactions were performed in 1× DUB assay buffer containing 1 mM dithiothreitol at 25°C, unless otherwise specified. The DUB activity assay kit was obtained from Cayman Chemical (Ann Arbor, MI, United States). For Ub-AMC assays, recombinant DUBs (rDUB; USP1/UAF1, USP1, USP2, or USP14) were pre-incubated with 1 μM of small molecule compounds (SJB2-043, ML-323, or PD) for 30 min. The reaction was started by adding Ub-AMC to a final concentration of 0.5 μM. After 30 min of incubation, the fluorescence of free AMCs in FLUOstar Omega microplate reader (BMG Labtech, Germany) was measured using excitation and emission wavelengths of 355 and 455 nm, respectively.

### Experimental Animals

Male BABL/C (8-week-old; body weight: 22–25 g) and C57/BL6J (7-week-old; body weight: 20–22 g) mice were purchased from DBL (Seoul, Korea). The animals were housed in a conventional state at an adequate temperature (23°C) and humidity (60%) with a 12/12 h light/dark cycle and provided with free access to water and food. The animals were acclimated to their environment for 5 days before being used in the experiments.

### Miles Assay

Experimental mice (BABL/C) were anesthetized and shaved. After 2–3 days, the mice were anesthetized again and intravenously injected with 100 μL of 1% Evans blue dye. After 15 min, intradermal injection of one of the following was performed: 50 μL of VEGF (50 ng/ml), histamine (500 nmol/L), primaquine diphosphate (1 or 10 μg), and PBS as a negative control. After 30 min, the back skin was photographed and dissected. The dye was then eluted from the dissected samples with formamide at 56°C, and the optical density was measured by spectrophotometry (FLUOstar Omega microplate reader) at 620 nm.

### Streptozotocin-Induced Diabetic Retinopathy Model

To induce diabetes mellitus, 60 mg/kg of STZ (Sigma-Aldrich) was injected intraperitoneally into 8-week-old C57/BL6J mice for five consecutive days. Body weights and glucose levels in tail vein blood samples were monitored weekly. One week after STZ injection, the mice were confirmed to be diabetic if their glucose level was greater than 300 mg/dl. These mice were divided into four groups: normal, STZ only, STZ + PD 0.5, and STZ + PD 1 mg/kg. PD was administered orally for 10 days, starting 4 weeks after STZ injection. One day after the last oral administration, FITC-dextran (70 kDa; Sigma-Aldrich) was injected into the heart and circulated in the body, followed by euthanization 5 min later. Both eyes of each mouse were used for examination of the retinal vascular pattern and were flat mounted on slides and analyzed using a confocal microscope (LSM 700 META; Carl Zeiss). Confocal images were quantified using Multi Gauge V2.2. The number of pixels in the leaked areas was compared with the total number of pixels in the entire retina.

### Statistical Analysis

Data are presented as mean ± standard error of the mean (SEM). All statistical analyses were performed using GraphPad Prism version 8 (GraphPad Software, La Jolla, CA, United States). Differences in means among the groups were statistically analyzed by one-way analysis of variance (ANOVA) with Tukey’s multiple comparison tests to elucidate leakage-related differences among experimental groups. Statistical significance was set at *p* < 0.05.

## Results

### Primaquine Diphosphate Blocks Vascular Endothelial Growth Factors-Induced Permeability in Human Umbilical Vein Endothelial Cells

**FIGURE 1:**
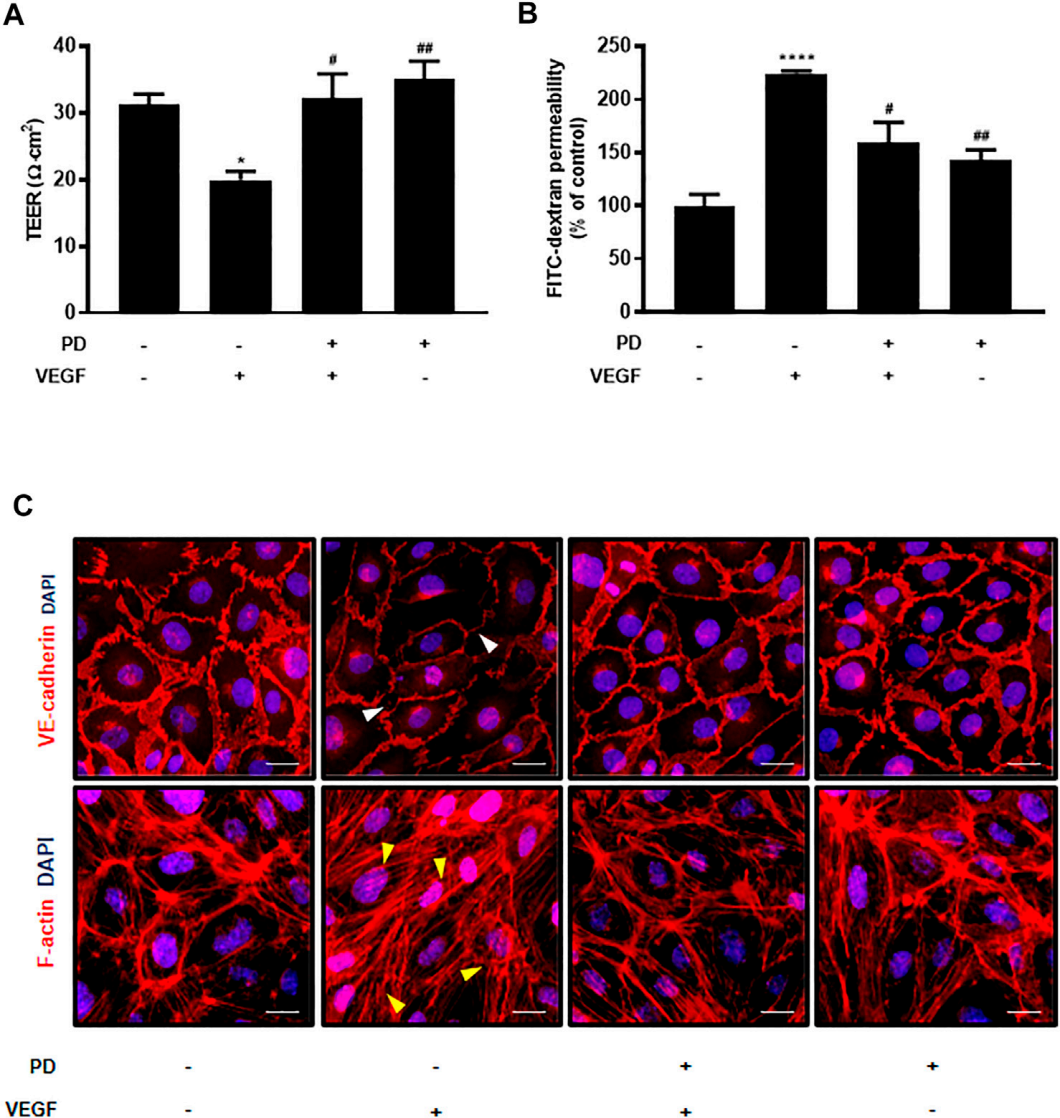
Primaquine diphosphate (PD) blocks VEGF-induced endothelial permeability and stabilizes junctional proteins. HUVECs were starved and treated with or without PD (5 μM, 30 min) before stimulation with VEGF (30 ng/ml, 30 min). PD blocked both TEER decline **(A)** and increased FITC-dextran transendothelial permeability **(B)** induced by VEGF. TEER was measured using Millicell ERS-2 (Millipore). For the permeability assay, FITC-dextran was added to the upper chamber. The absorbance of the solution in the lower chamber was measured at 492 nm (excitation) and 520 nm (emission) using a FLUOstar Omega microplate reader. *n* ≥ 3 independent experiments. **(C)** HUVECs were starved and treated with or without PD (5 μM, 30 min) before stimulation with VEGF (50 ng/ml, 30 min). Cells were fixed, permeabilized, and immunostained for VE-cadherin and F-actin. White arrow indicates an attenuated VE-cadherin expression and yellow arrow shows stress fiber formation. All data are presented as means ± SEM, *p < 0.05, ****p < 0.0001 vs. control group; #*p* < 0.05, ##*p* < 0.005 vs. VEGF treatment group. Scale bar = 20 μM.

We tested 1,018 FDA-approved drugs to find repositioning drugs as vascular leakage blockers. We first screened drugs that have a protective effect on endothelial cells. Among these, drugs that have not yet been studied in blood vessels were secondarily screened, and PD, a drug involved in junction stabilization, was discovered through *in vitro* assays (Supplementary Figure S1A). To evaluate the protective effect of PD on endothelial barrier integrity, the potential changes in the integrity of endothelial cells were assessed by measuring the TEER and permeability of huvec monolayers to FITC-dextran. PD blocked TEER reduction and FITC-dextran leakage caused by VEGF treatment (Figures 1A,B). In addition, we tested the effect of PD on the stability of the AJ protein VE-cadherin and expression of F-actin by immunostaining. Normally, confluent HUVECs display a linear pattern of AJ proteins at the cell borders, and this characteristic localization is disrupted by VEGF (Oldenburg and de Rooij, 2014; van Buul and Timmerman, 2016). PD inhibited the disruptive effect of VEGF treatment by preventing the linear distribution of VE-cadherin (Figure 1C). Furthermore, F-actin immunostaining showed that in the control, confluent HUVECs had ring-like shapes (Bijman et al., 2006). VEGF treatment disrupted cortical actin ring structures and increased actin stress fiber formation whereas PD significantly prevented VEGF-induced stress fiber formation and maintained the cortactin ring shape (Figure 1C). Collectively, these results demonstrate that PD has a barrier protective effect by stabilizing AJ proteins and the actin cytoskeleton.

### Primaquine Diphosphate Exhibit Reduced Vascular Permeability in Response to Vascular Endothelial Growth Factors and Histamine

**FIGURE 2:**
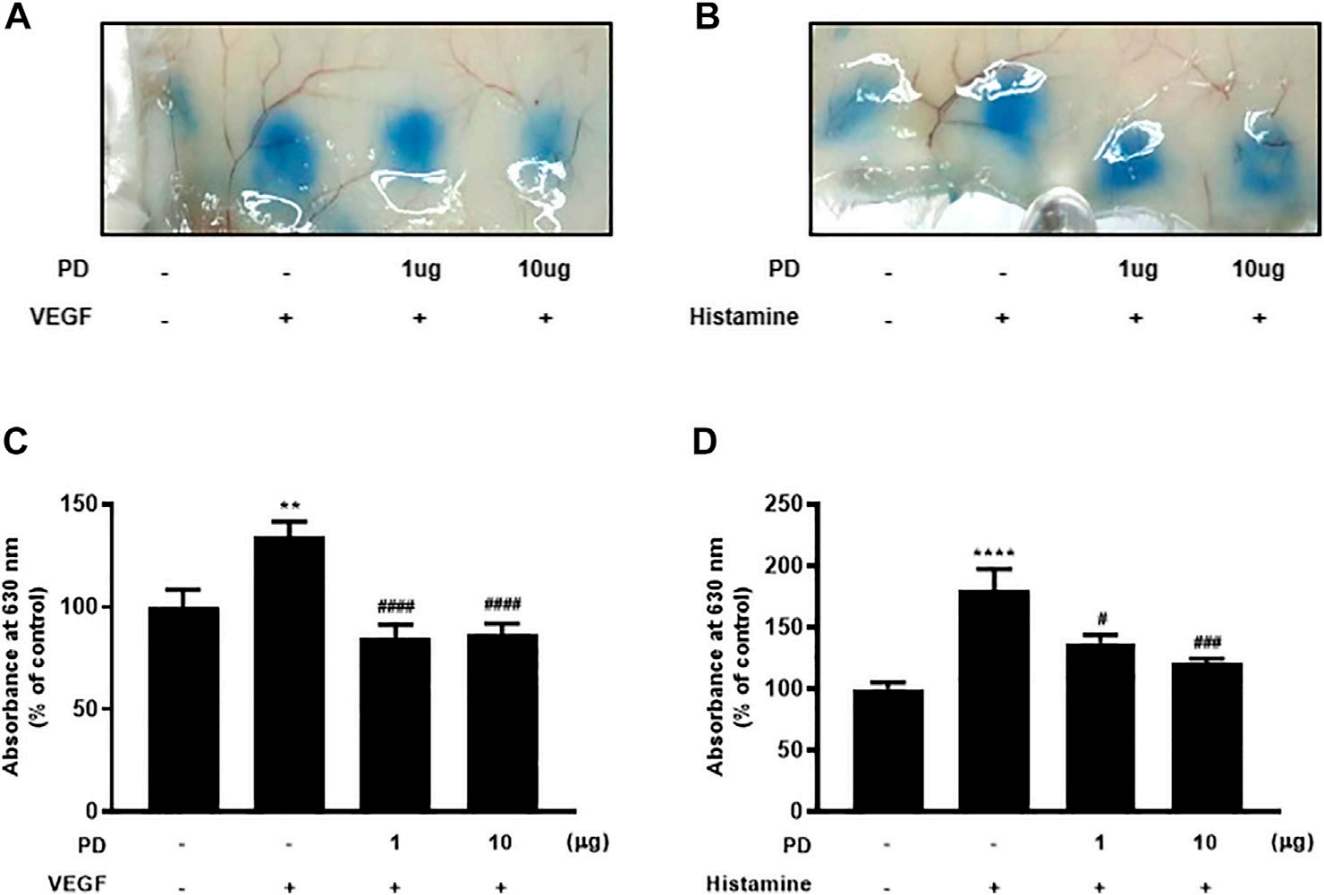
PD treatment blocks VEGF-induced and histamine-induced permeability *in vivo*. Vascular permeability was assessed using the Miles assay. Six-week-old mice were used, and mice were tail vein-injected with Evans blue dye followed by intradermal injection of VEGF (50 ng), histamine (500 nM), or PD (1 μg or 10 μg) **(A,B)**. The skin at the injection site was collected and photographed. **(C,D)** Evans blue dye was extracted from the skin by incubation with formamide at 56°C overnight, and the absorbance of the extracted dye was measured at 620 nm with a spectrophotometer (*n* = 7). All data are presented as means ± SEM, *p < 0.005, ****p < 0.0001 vs control group; #*p* < 0.05, ###*p* < 0.0005, ####*p* < 0.0001 vs inducer treated group.

The Miles assay was performed using VEGF and histamine to evaluate whether PD regulates vascular permeability *in vivo*. The Miles assay is a commonly used, well-established, and relatively simple technique that measures vascular leakage *in vivo* as a measure of vascular hyperpermeability (Brash et al., 2018). Several permeable factors such as VEGF and histamine have been demonstrated to compromise vascular homeostasis (Feng et al., 1996). The backs of mice, into which permeability factors were injected, turned blue owing to the extravasation of Evans blue dye, which had been systemically administered. The extravasation of Evans blue induced by VEGF was reduced by PD (Figures 2A,C). In addition, dermal injection of histamine led to vascular leakage of Evans blue dye, which was blocked by PD in a dose-dependent manner (Figures 2B,D). Taken together, these results suggest that VEGF and histamine-induced vascular permeability are suppressed by PD.

### Primaquine Diphosphate Effectively Diminished Retinal Vascular Leakage in a Diabetic Mouse Model

**FIGURE 3:**
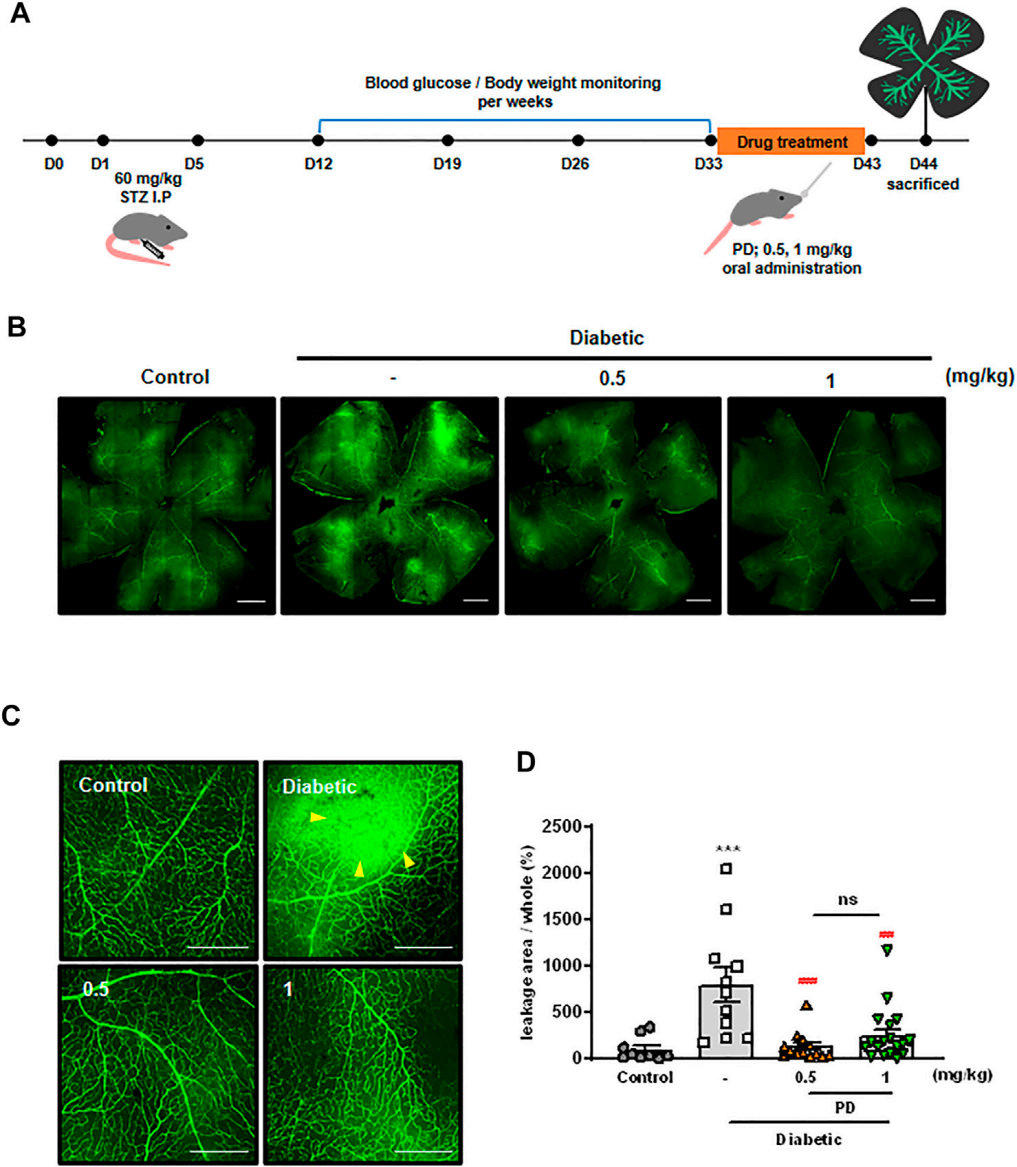
Oral administration of PD attenuates diabetes-induced vascular retinal leakage. **(A)** Scheme of PD treatment on STZ-induced diabetic mouse model. Diabetic mice were treated with various concentrations of PD (0.5 and 1 mg/kg) for 10 days by oral gavage; 24 h after the last treatment, 200 μL FITC-Dextran (30 mg/ml in sterile PBS) was injected into the left ventricle. Retinas were viewed using confocal microscopy. **(B)** Representative images for fluorescein angiography from the control, untreated DM, and PD-treated DM groups at 4 weeks after STZ injection are shown at ×100 magnification. **(C)** Representative images are enlarged images in **(B)**, ×200 magnification. Yellow arrowheads indicate the region of vascular permeability. **(D)** The quantified statistical analysis of the area of FITC-dextran leakage in **(B)** is shown by multi-gauge software (Fuji). Data are the mean ± SEM. ***p < 0.0005 vs control group; ###*p* < 0.0005, ####*p* < 0.0001 vs vehicle group (n > 9). Scale bar = 500 μM.

Disruption of junctions in endothelial cells leads to vascular leakage and fluid exudation into the surrounding tissue, which can lead to serious diseases such as DR (Bandello et al., 2013; Claesson-Welsh et al., 2020). To evaluate the effect of PD on DR, STZ-induced diabetic mice were orally administered with PD, and retinal vascular leakage was investigated using immunofluorescence staining (Figure 3A). High levels of extravasation of FITC-dextran were observed in the retinas of diabetic mice, and this vascular leakage was blocked by oral administration of PD. Measurement of FITC-dextran in the retina showed that 0.5 mg/kg PD effectively reduced retinal vascular leakage in a STZ-induced diabetic mouse model (Figures 3B,C). PD prevention against vascular leakage in the retinas of diabetic mice was quantitatively analyzed by determining the leakage area of FITC-dextran in whole retinal tissues (Figure 3D). Taken together, these findings show that PD helped prevent retinal vascular leakage in diabetic mice by inhibiting permeability by VEGF and inflammatory cytokines such as histamine.

### Ubiquitin Specific Protease 1 Regulates the Integrity of the Endothelial Barrier as a Target of Primaquine Diphosphate

**FIGURE 4:**
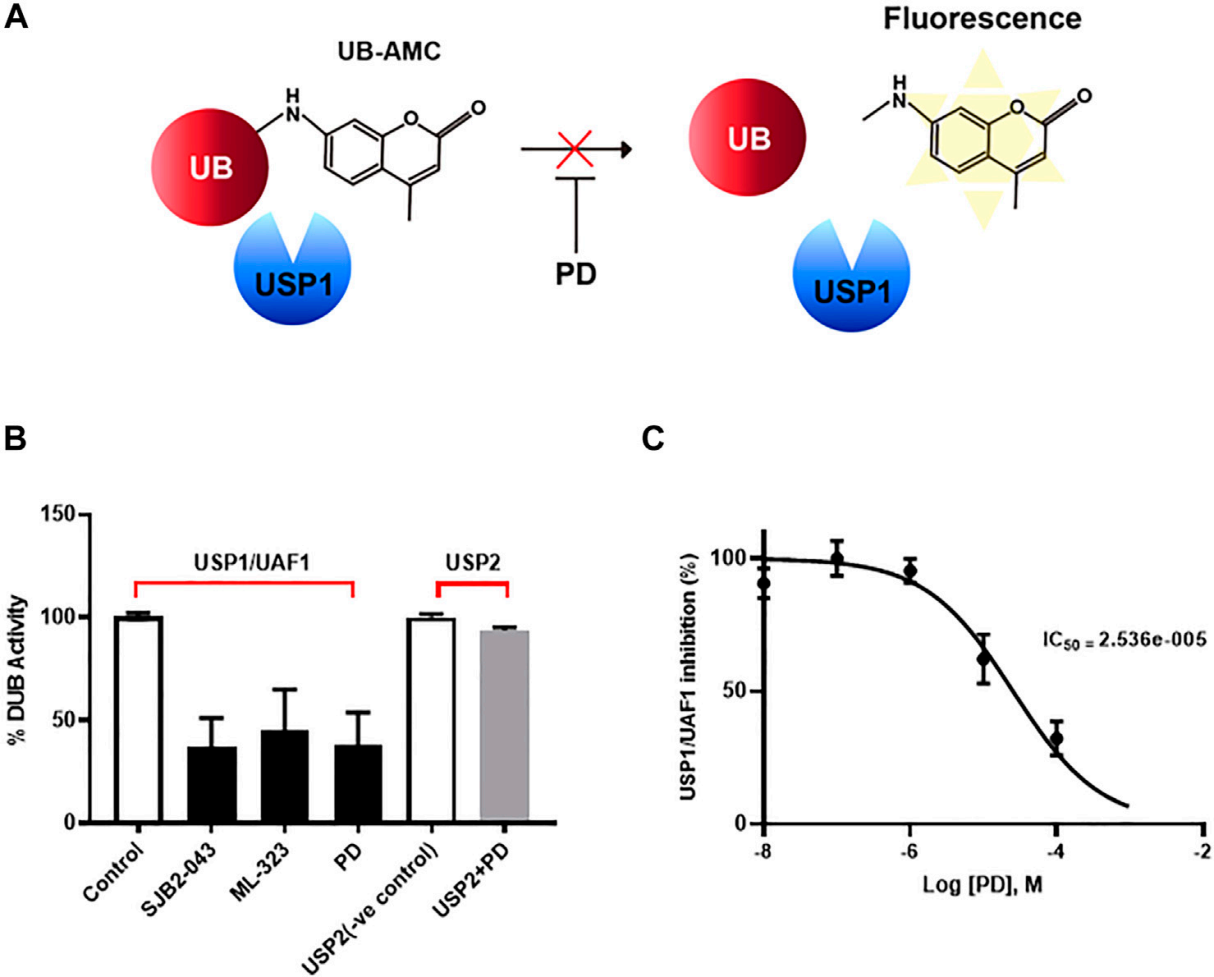
PD specifically inhibited USP/UAF1 activity. **(A)** Schematic representation of Ub-AMC assay. USP1 removes ubiquitin from its substrate Ub-AMC, and fluorescent AMC is measured. **(B)** rUSP1/UAF1 complex or rUSP2 were incubated with control, USP1 inhibitor SJB2-043, ML-323, or PD for 30 min at 37°C, followed by assessment of DUB activity using Ub-AMC assay. **(C)** Progress curve for USP1/UAF1 activity on PD against Ub-AMC. The graph represents the average of three independent experiments with calculated SEM. The experiment was conducted at least four times.

**FIGURE 5:**
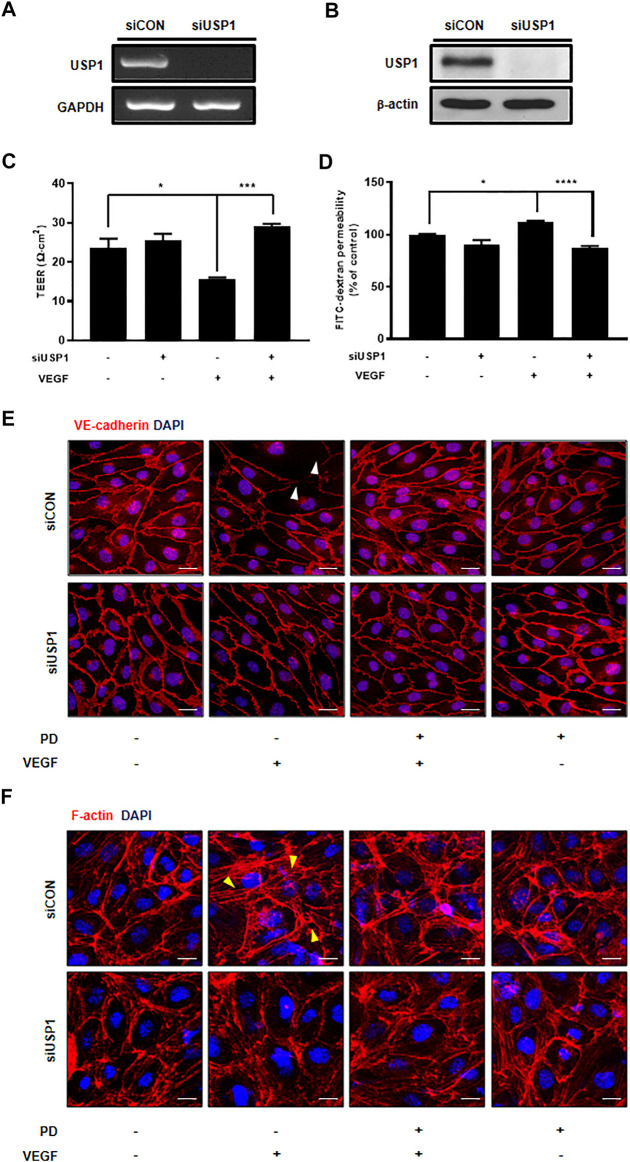
USP1 knockdown inhibits VEGF-induced endothelial permeability and stabilizes junctional proteins. **(A)** Expression of USP1 mRNA by RT-PCR following transfection with USP1 siRNA. **(B)** Western blot analysis shows USP1 expression in cells transfected with USP1-siRNA. Treatment with both VEGF and siUSP1 resulted in decreased endothelial permeability of TEER **(C)** and 70 kDa dextran **(D)** as compared with that obtained with VEGF treatment alone in HUVECs. *n* ≥ 3 independent experiments. siUSP1 cells were fixed, permeabilized, and subsequently immunostained for VE-cadherin **(E)** and F-actin **(F)**. All data are presented as means ± SEM. *p < 0.05, ***p < 0.0005, ****p < 0.0001. Scale bar = 20 μM.

**FIGURE 6:**
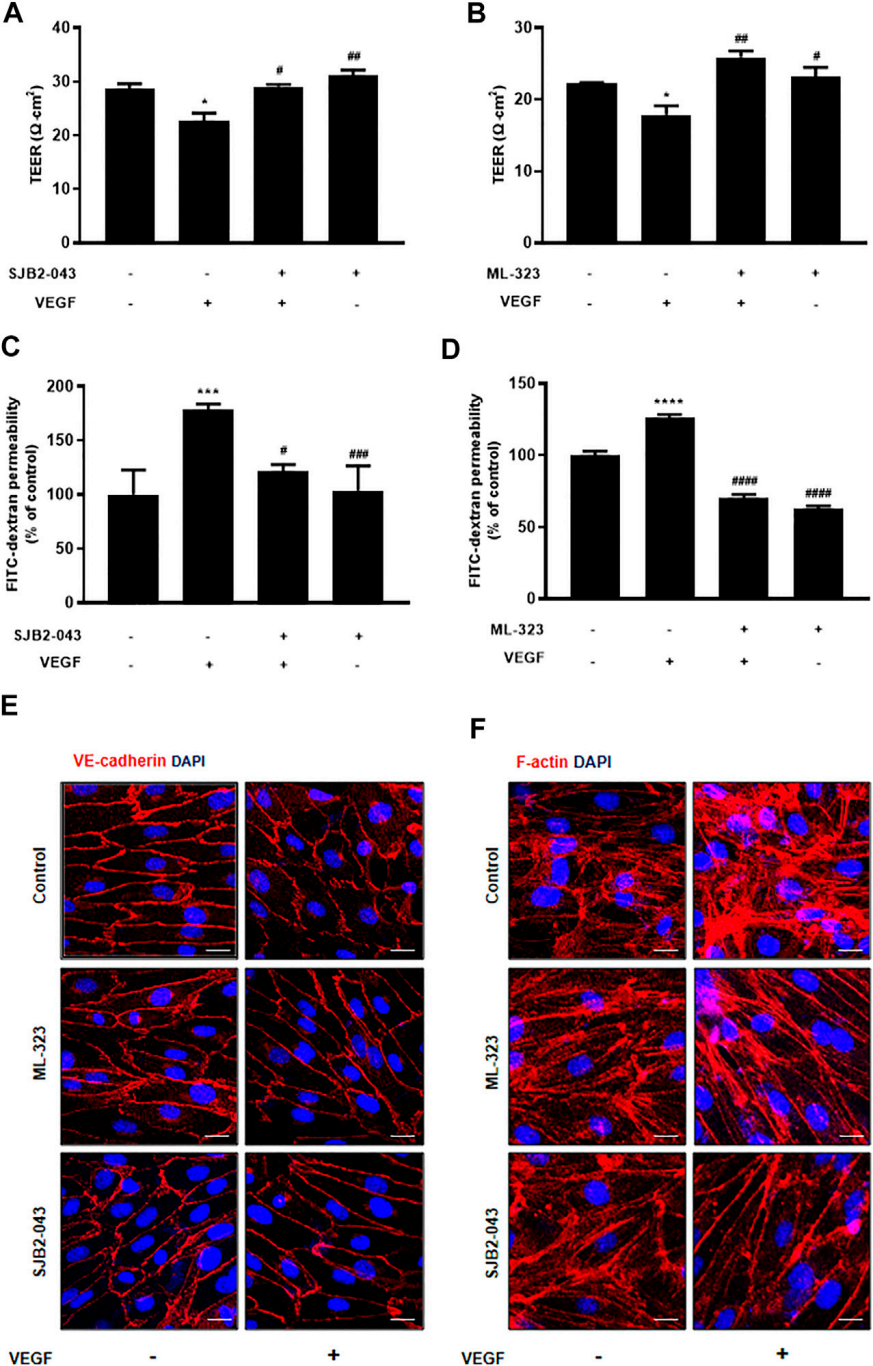
USP1 inhibitor blocks VEGF-induced endothelial permeability and stabilizes junctional proteins. HUVECs were starved and treated with SJB2-043 (1 μM, 30 min) and ML-323 (5 μM, 30 min) before stimulation with VEGF (30 ng/ml, 30 min). SJB2-043 and ML-323 blocked both the TEER decline **(A,B)** (Experiments were performed in triplicate) and the increase in FITC-dextran transendothelial permeability **(C,D)** (Combined data from the six independent experiments are shown) induced by VEGF. HUVECs were treated with SJB2-043 (1 μM, 30 min) and ML-323 (5 μM, 30 min) before stimulation with VEGF (50 ng/ml, 30 min). Cells were fixed, permeabilized, and subsequently immunostained for VE-cadherin **(E)** and F-actin **(F)** All data are presented as means ± SEM, *p < 0.05, ***p < 0.0005, ****p < 0.0001 vs control group; #*p* < 0.05, ##*p* < 0.005, ###*p* < 0.0005, ####*p* < 0.0001 vs VEGF treated group. Scale bar = 20 μM.

Although PD has been demonstrated to prevent vascular leakage, the target in endothelial cells remains unclear (Camarda et al., 2019). To identify the mode of action for PD that stabilizes the vascular barrier, we identified targets through prediction programs and the Ub-AMC assay. Target prediction programs were used to identify the common targets (Supplementary Figure S2A). Among these targets, USP1 was highly expressed in endothelial cells (Supplementary Figure S2B). We evaluated the effect of PD on USP1 or other DUBs by using the Ub-AMC (ubiquitin 7-amino-4-methylcoumarin) assay (Figure 4A, Supplementary Figure S2C). An *in vitro* assay with purified rDUB showed that PD inhibited USP1/UAF1 activity. Importantly, PD did not significantly affect USP2 activity (Figure 4B). PD inhibits USP1/UAF1 activity with an IC50 value of 2.536e-005 M (Figure 4C). To examine whether USP1 is the target of PD, we induced loss of function of USP1. Reverse transcription-PCR (RT-PCR) and western blotting showed a significant decrease in USP1 levels compared with those in controls 48 h after transfection with USP1-knockdown siRNA in HUVECs (Figures 5A,B). USP1 knockdown also significantly increased TEER values and markedly reduced leakage of FITC-dextran compared with these in the control group, indicating that this prevented VEGF-induced permeability (Figures 5C,D). Furthermore, silencing of USP1 was able to prevent VEGF-induced disruption of VE-cadherin and cortical actin ring formation (Figures 5E,F). To further confirm the role of USP1 in maintaining vascular barrier integrity, we performed a permeability assay using two USP1 inhibitors, SJB2-043 and ML-323. The TEER assays demonstrated that SJB2-043 and ML-323 prevented VEGF-induced permeability (Figures 6A,B), and the same effect was also observed in FITC-dextran permeability assay (Figures 6C,D). In addition, treatment with USP1 inhibitors protected VEGF-induced disruption of VE-cadherin and actin stress fiber formation (Figures 6E,F). Taken together, these data demonstrate that PD regulation of USP1 reduces permeability and maintains barrier integrity in VEGF-induced endothelial cells.

## Discussion

Regulation of endothelial barrier function is critical for vascular function and integrity (Lum and Malik, 1994; Surapisitchat and Beavo, 2011). Vascular barrier integrity can be destroyed by various soluble permeability factors, and changes in barrier function during disease progression can exacerbate tissue damage. Restoration of the normal vascular structure is believed to reduce hyperpermeability (Park-Windhol and D’Amore, 2016). Here, we discovered PD, a new vascular leakage blocker, and confirmed that this drug inhibited endothelial permeability by stabilizing AJs and attenuating vascular leakage.

The maintenance of endothelial cell integrity is regulated by cytoskeletal tissue and intercellular junctions, such as AJs and TJs (Popoff and Geny, 2009). Several studies have shown that AJ protein dissolves when treated with a permeable factor (Bates et al., 1999; Moy et al., 2000; Dvorak, 2002; Shasby et al., 2002; Fu and Shen, 2003; Adam, 2015). VEGF induces actin stress fiber formation and leads to vascular permeability (Rousseau et al., 2000). In addition, the dissolution of cortactin by permeability factors causes the formation of actin stress fibers and destabilizes barrier integrity (Oldenburg and de Rooij, 2014; van Buul and Timmerman, 2016). Our study used permeability assays and the expression pattern of junctional proteins by immunostaining to demonstrate that PD treatment in VEGF-induced endothelial cells reduced leakage. PD treatment prevented VEGF-induced degradation of VE-cadherin, which resulted in reduced permeability of HUVECs. We further showed that PD blocked VEGF-induced actin stress fiber formation in HUVECs by reorganizing actin into the cortical actin ring. Altogether, these data suggest that PD has the potential to reorganize the dispersed actin polymers into cortical actin rings with subsequent stabilization of AJs, which may be responsible for endothelial barrier enhancement. In addition, we confirmed that PD prevented VEGF-and histamine-induced vascular permeability in the Miles assay. VEGF, a vascular permeability factor, is a major pathogenic molecule involved in the occurrence of complications (i.e., diabetic and hypertensive retinopathy, age-related macular degeneration) (Apte et al., 2019), and histamine is a representative inflammatory mediator that strongly induces blood vessels and permeability. Several studies have shown increased VEGF and histamine expression in diabetic retina (Lee et al., 2020). One therapeutic strategy for reducing these permeability aims to develop a compound that can stabilize endothelial cell junctions, which are disrupted in the disease (Stylianopoulos and Jain, 2013; Tan et al., 2015). A STZ-induced diabetic mouse model was shown to have increased retinal vascular leakage similar to that observed in the early stage of human DR (Su et al., 2000; Yu et al., 2001). Here, oral administration of PD significantly decreased retinal vascular leakage in a diabetic mouse model. Our data suggest that PD can reduce vascular leakage in DR by blocking multiple factors. PD is the most representative member of antimalarial 8-aminoquinoline (Vale et al., 2009; Ashley et al., 2014), which show remarkable activity against gamete cells of all species of human malaria, including the multi-resistant *Plasmodium falciparum* strain (Vale et al., 2009; Vale et al., 2014). This drug eliminates the malaria parasites living in other body tissues, preventing the occurrence of red blood cell forms of the parasites that cause relapses in P. vivax infection and malaria (White, 2011). New chemotherapy strategies can now be devised after the recent discovery that gamete formation in malaria parasites is mediated by the cGMP-dependent protein kinase PKG (McRobert et al., 2008). However, despite these findings, the definite mode of action of PD has been elusive for decades, and its role in endothelial cells remains unclear.

Thus, in this study, we further explored the possible mechanisms of PD in the regulation of barrier function. A target prediction program was used to identify the common targets of PD. Of these targets, we decided to investigate the regulation of USP1, which is highly expressed in endothelial cells. Interestingly, as assessed in the DUB activity assay, PD played a role similar to that of an inhibitor of USP1. In addition, USP1 knockdown and inhibition reduced the VEGF-induced permeability and stabilized integrity. Today, ubiquitination is recognized as a key factor in regulating the overabundance of cellular functions and plays an important role in cellular homeostasis (Reyes-Turcu et al., 2009; Garcia-Santisteban et al., 2013). USPs act on specific proteins, and therefore controlling them can improve prognosis (da Silva et al., 2009; Sacco et al., 2010; Schwickart et al., 2010; Tavana and Gu, 2017). For example, the proteasome inhibitor bortezomib improves myocardial ischemia/reperfusion injury, prevents post-ischemic ventricular tachyarrhythmia, promotes cardiac hypertrophy regression, and reverses vascular endothelial dysfunction caused by diabetes (Raimondi et al., 2019; Bencsik et al., 2020). In addition, inhibition of USP1 has the potential to target a variety of cancers (Sacco et al., 2010; Chen et al., 2011; Ma et al., 2019). According to recent studies, USP1 contributes to deubiquitination and stabilization of the differentiation inhibitory proteins of the DNA binding inhibitor (ID) family, and ID1 is known to be associated with permeability (Zhang et al., 2015; Lee et al., 2016; Gadomski et al., 2020). As the expression of ID1 regulates E-cadherin expression (Li et al., 2007), USP1 is likely to contribute the stabilization of adherens junction protein via ID1 in endothelial cells. Taken together, our findings suggest that USP1 stabilizes endothelial cells from mediators of increased endothelial permeability. Further investigation is needed to clarify USP1 function and regulation of the endothelial barrier.

We used drug repositioning and laboratory screening systems to identify that PD can act as a vascular leakage blocker. Drug repositioning is a strategy to identify new uses of drugs approved to treat conditions that are different from their original purpose (Benavides-Cordoba, 2020). This strategy can deliver results with an improved level of safety and lower cost in a shorter time (Xue et al., 2018). We tested the viability of 1,018 FDA-approved drugs in the serum-free state, and an *in vitro* assay confirmed that PD has the potential to reconstitute the dispersed actin rings and subsequent stabilization of the adhesion junction, which can strengthen the endothelial barrier. The molecular mechanisms that control vascular leakage have been studied for decades, and more recently, the focus has been on identifying treatment-related agents that further restrict fluid and solute exchange by targeting the endothelial barrier directly. Application of the existing knowledge of endothelial barrier regulation is necessary to develop therapeutic agents that can be used routinely to protect or enhance endothelial barrier function. Our study indicates that PD effectively prevents barrier permeability. Thus, PD could be therapeutically used for vascular leakage diseases, such as diabetic retinopathy, ischemic stroke, and chronic inflammatory diseases related to endothelial barrier dysfunction.

## Data Availability

The original contributions presented in the study are included in the article/Supplementary Material, further inquiries can be directed to the corresponding author.
