# Supplementary material for: Primaquine Diphosphate, a Known Antimalarial Drug, Blocks Vascular Leakage Acting Through Junction Stabilization
Source: Front Pharmacol. 2021 Jun 4;12:695009. doi: 10.3389/fphar.2021.695009 (PMC8211987; doi:10.3389/fphar.2021.695009)
Supplement: Supplementary file 1 [file DataSheet1.docx]

**Supplementary figures and figure captions**

**
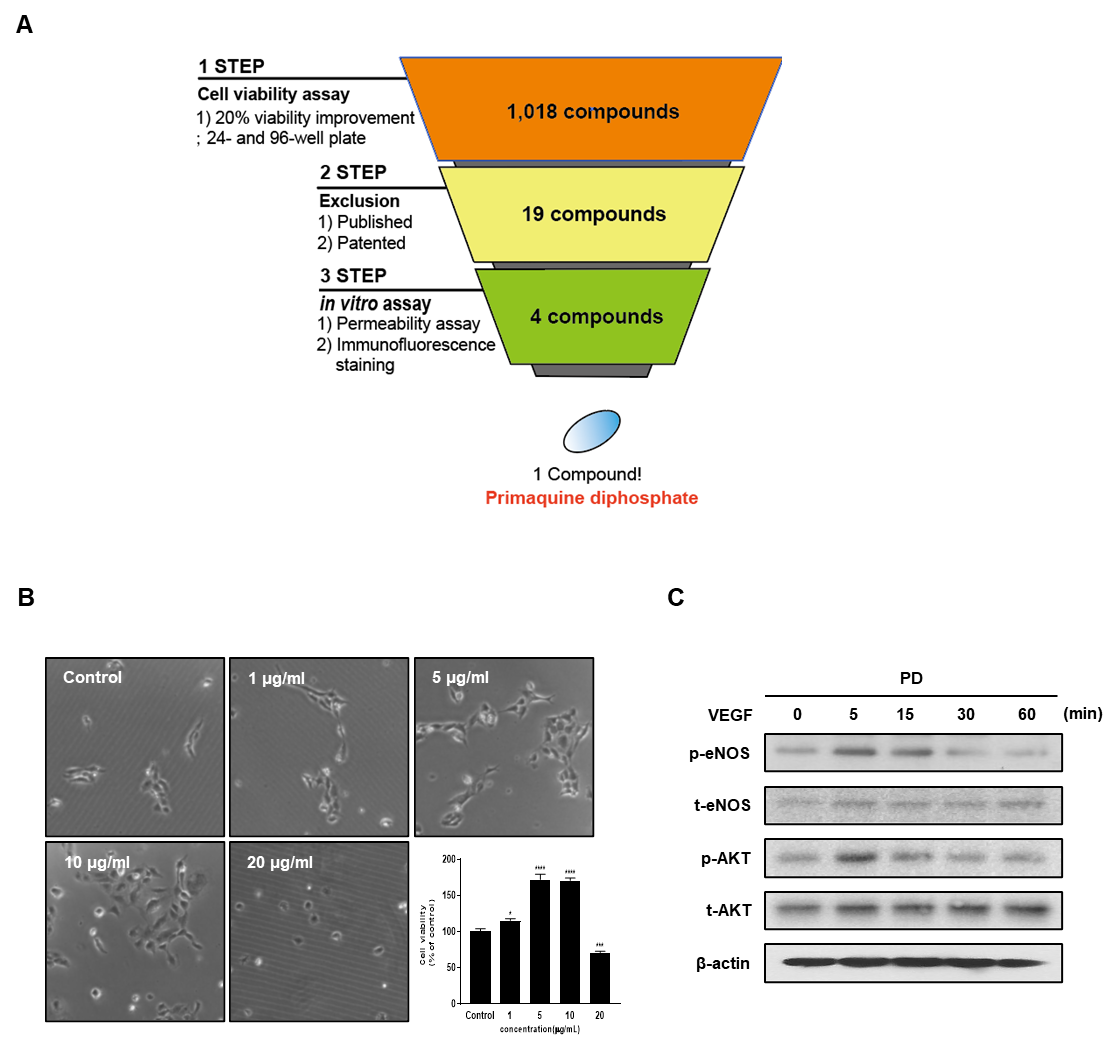
**

**Supplementary Figure 1.**

**Drug screening for a new vascular leakage blocker**. (**A**) The concept of drug repositioning approach for finding new indications from existing drugs for vascular leakage blocker. (**B**) PD increases HUVECs survival. HUVECs were starved and treated for 48 h with various concentrations of PD. Cell viability significantly increased with dose-dependent. (**C**) The effects of PD on Akt-eNOS pathway in HRECs. PD causes a time-dependent phosphorylation of Akt at Ser473 and eNOS at Ser1177 in HRECs. Data are the mean ± SEM. *P < 0.05, **P < 0.005, ***P < 0.0005.


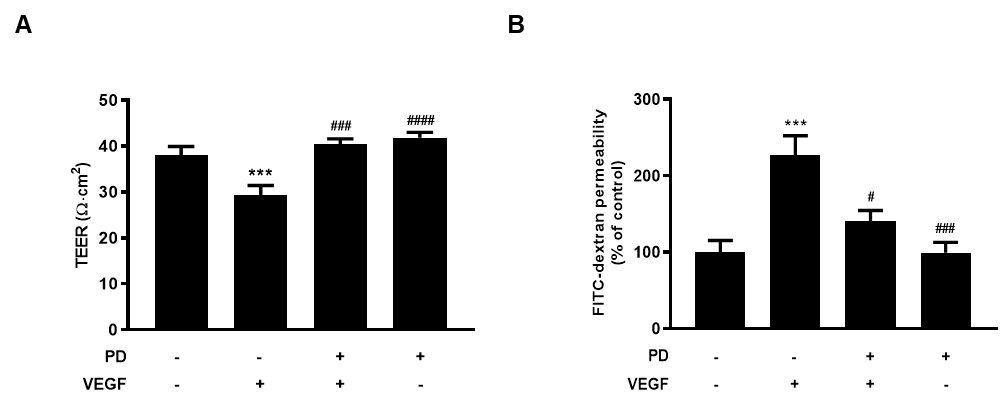


**Supplementary Figure 2.**

**Primaquine diphosphate (PD) blocks VEGF-induced permeability in HRECs**. HRECs were starved and treated with or without PD (5 µM, 30 min) before stimulation with VEGF (30 ng/mL, 30 min). PD blocked both TEER decline (**A**) and increased FITC-dextran transendothelial permeability (**B**) induced by VEGF. TEER was measured using Millicell ERS-2 (Millipore). For the permeability assay, FITC-dextran was added to the upper chamber. The absorbance of the solution in the lower chamber was measured at 492 nm (excitation) and 520 nm (emission) using a FLUOstar Omega microplate reader. ​n = 3 independent experiments. All data are presented as means ± SEM, ***P < 0.0005 vs control group; #P < 0.05, ###P < 0.0005, ####P < 0.0001 vs inducer treat group.


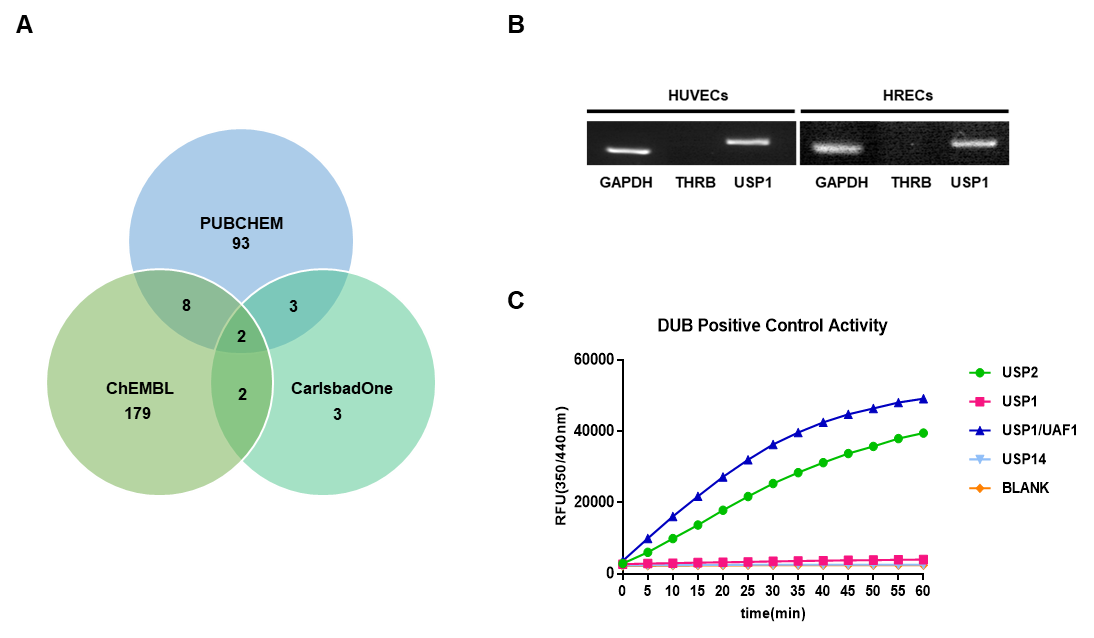


**Supplementary Figure 3.**

**Discovering the mechanism of PD**. (**A**) Venn diagram of PD targets determined by target prediction software (PUBCHEM: https://pubchem.ncbi.nlm.nih.gov/, CheMBL: https://www.ebi.ac.uk/chembl/, Carlsbadone: https://datascience.unm.edu/carlsbad/). Only two genes were predicted to be targets of PD by all three programs, including USP1. (**B**) Expression levels of THRB and USP1 measured by qPCR in HUVECs and HRECs. (**D**) The Ub-AMC (ubiquitin 7-amino-4-methylcoumarin) assay was used to assess the activity of several USP families.


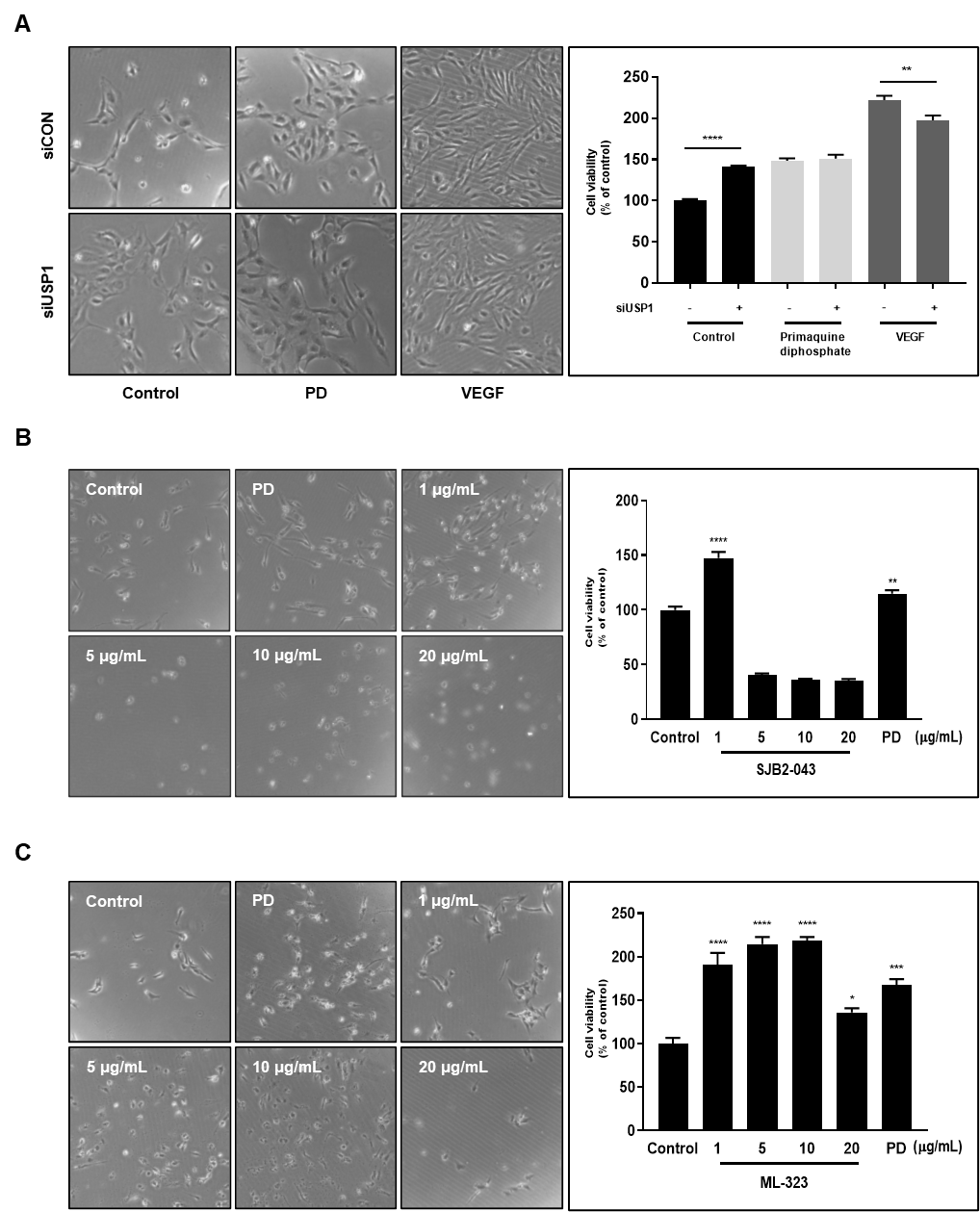


**Supplementary Figure 4.**

**Cell survival was detected using an MTT assay**. (**A**) Scr-siRNA or USP1-siRNA-transfected endothelial cells were starved and treated with PD and VEGF. USP1 knockdown increases HUVEC survival. (**B**, **C**) HUVECs were starved and treated with various concentrations of USP1 inhibitors: SJB2-043 and ML-323. MTT assay was performed 48 h post-incubation to measure the cell viability. All data are presented the means ± SEM. **P < 0.005, ***P < 0.0005, ****P < 0.0001.
